# Reliability of different methodologies of infrared image analysis of myofascial trigger points in the upper trapezius muscle

**DOI:** 10.1590/bjpt-rbf.2014.0076

**Published:** 2015-04-27

**Authors:** Almir V. Dibai-Filho, Elaine C. O. Guirro, Vânia T. K. Ferreira, Hugo E. Brandino, Maíta M. O. L. L. Vaz, Rinaldo R. J. Guirro

**Affiliations:** Programa de Pós-graduação em Reabilitação e Desempenho Funcional, Departamento de Biomecânica, Medicina e Reabilitação do Aparelho Locomotor, Faculdade de Medicina de Ribeirão Preto, Universidade de São Paulo (USP), Ribeirão Preto, SP, Brazil

**Keywords:** myofascial pain syndromes, thermography, skin temperature, physical therapy

## Abstract

**BACKGROUND:**

Infrared thermography is recognized as a viable method for evaluation of subjects with myofascial pain.

**OBJECTIVE:**

The aim of the present study was to assess the intra- and inter-rater reliability of infrared image analysis of myofascial trigger points in the upper trapezius muscle.

**METHOD:**

A reliability study was conducted with 24 volunteers of both genders (23 females) between 18 and 30 years of age (22.12±2.54), all having cervical pain and presence of active myofascial trigger point in the upper trapezius muscle. Two trained examiners performed analysis of point, line, and area of the infrared images at two different periods with a 1-week interval. The intra-class correlation coefficient (ICC_2,1_) was used to assess the intra- and inter-rater reliability.

**RESULTS:**

With regard to the intra-rater reliability, ICC values were between 0.591 and 0.993, with temperatures between 0.13 and 1.57 °C for values of standard error of measurement (SEM) and between 0.36 and 4.35 °C for the minimal detectable change (MDC). For the inter-rater reliability, ICC ranged from 0.615 to 0.918, with temperatures between 0.43 and 1.22 °C for the SEM and between 1.19 and 3.38 °C for the MDC.

**CONCLUSION:**

The methods of infrared image analyses of myofascial trigger points in the upper trapezius muscle employed in the present study are suitable for clinical and research practices.

## Introduction

Myofascial trigger points are structures found in skeletal muscles that present with some type of dysfunction. Conceptually, they are nodules hypersensitive to palpation due to pain, change in muscular activity, limitation in joint mobility, and autonomic manifestations^1^. In addition, the literature suggests that the physiopathological mechanism of myofascial trigger points is related to changes in muscular activity and the repercussions for blood circulation and local metabolism^2^^,^ 3.

Myofascial trigger points may present as active or latent. The active points are hypersensitive points that trigger pain recognized as familiar by the patient during the application of compressive force; in turn, latent points are clinically quiescent with respect to spontaneous pain, generating unfamiliar pain^2^. The active myofascial trigger points also differ by the presence of different algesic substances, such as bradykinin, substance P, and serotonin^3^.

Recent studies^4^^-^ 6 have used the criteria set by Simons et al.^7^ for the correct diagnosis of myofascial trigger points based on muscle palpation. However, other authors highlight that palpation requires a combination of skill, training, and critical clinical practice^8^. In addition, other instruments may be applied for evaluation of subjects with myofascial trigger points, such as ultrasonography^9^, sonoelastography^10^, and electromyography^11^. However, despite advances in diagnostic technology, physical examination remains the most accepted method of evaluation of myofascial trigger points due to the limited clinical applicability of the new instruments.

Within this context and considering both autonomic and metabolic repercussions resulting from the presence of myofascial trigger points^2^, infrared thermography is recognized as another viable method for the evaluation of subjects with myofascial pain, according to studies conducted by Hakgüder et al.^12^ and Haddad et al.^13^. This is a non-invasive method for evaluating the behavior of body skin temperature^14^, which is dependent on microcirculatory, metabolic, and autonomic activities^15^^,^ 16.

In general, infrared images can be evaluated in two ways: qualitatively, in which an experienced examiner gives an opinion based on the visual analysis of the image^17^^,^ 18; and quantitatively, in which body skin regions of interest are measured by means of specific software. According to the literature, the latter is the most used form^13^^,^ 19 ^-^ 21. However, despite the studies using infrared thermography in subjects with myofascial pain, there is a lack of standardization in the method of infrared image analysis, as reported by Costa et al.^21^.

In light of this, the objective of the present study was to assess the intra- and inter-rater reliability of infrared image analyses of myofascial trigger points in the upper trapezius muscle. The hypothesis tested herein is that the methodologies for the analysis of infrared images show reliability that endorses its use in clinical practice and research.

## Method

### Sample

A sample size calculation was performed with a confidence coefficient of 0.95 and a range of the confidence interval (CI) for the intraclass correlation coefficient (ICC) of 0.30. Fleiss's^22^ coefficients were also calculated to detect substantial reliability (ICC=0.76)^22^. Therefore, a sample size of 24 volunteers was estimated. The sample size calculation was based on the study conducted by Bonett^23^.

The target population of this study was recruited from the university community of Ribeirão Preto, SP, Brazil, by means of verbal invitation and posters. The inclusion criteria were the following: age group between 18 and 30 years old; both genders; presence of neck pain^24^, anatomically defined as pain within the region limited by the superior nuchal line, the lateral margins of the cervical vertebrae and an imaginary transverse line immediately above the first thoracic spinous process^25^, which was identified by a Neck Disability Index (NDI) score ≥5 points^26^^,^ 27 and a score ≥3 points according to the Numeric Pain Rating Scale^28^; use of computer for at least 2 hours daily^29^; and the presence of active myofascial trigger point, unilateral and of central location^30^ in the upper trapezius muscle on the same side of the dominance of the upper limb.

The diagnosis of the myofascial trigger point was performed only once according to the criteria established by Simons et al.^7^ and Gerwin et al.^31^: 1) presence of a palpable taut band in a skeletal muscle, 2) presence of a hypersensitive tender spot within the taut band, 3) local twitch response elicited by the snapping palpation of the taut band, and 4) reproduction of referred pain in response to myofascial trigger point compression. These criteria were found to have good levels of inter-rater reliability^31^. Myofascial trigger point was considered active if local and spontaneous pain evoked by digital compression was recognized as familiar pain by the volunteer^32^.

The exclusion criteria were the following: volunteers with history of cervical trauma; surgery of the head, face or neck; cervical disc disease; degenerative diseases of the spine; physical therapeutic treatment in the past 3 months; use of analgesics, anti-inflammatories or muscle relaxants in the past week; presence of systemic diseases; diagnosis of fibromyalgia; body mass index (BMI) greater than 25 kg/m^2^.

The procedures of the present study were approved by the Research Ethics Committee of Hospital das Clínicas da Faculdade de Medicina de Ribeirão Preto da Universidade de São Paulo (USP), Ribeirão Preto, SP, Brazil, according to protocol number 030643/2013. Each volunteer signed a consent form.

### Infrared thermography

**Figure 1.**
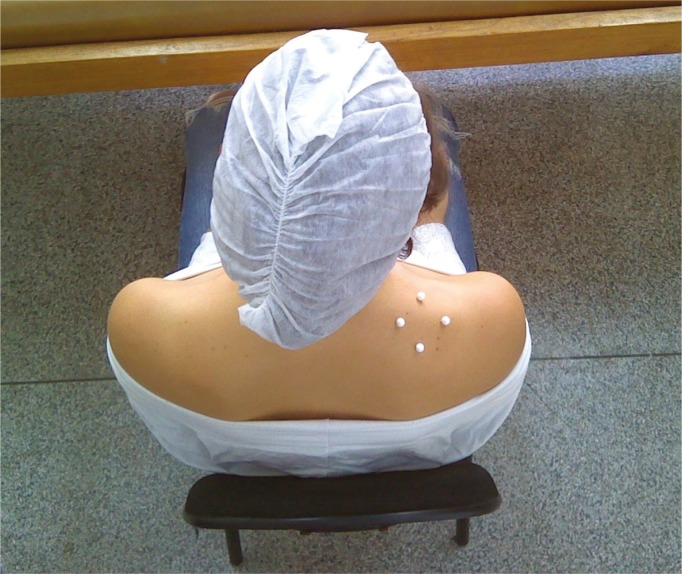
Styrofoam markers used to delineate the myofascial trigger point in the upper trapezius muscle.

Myofascial trigger points do not show a pattern of identification when analyzed by infrared imaging. Therefore, to ensure that the skin temperature was measured precisely on the myofascial trigger points, we initially performed palpation and identification of the myofascial trigger point centrally located^30^ in the upper trapezius muscle according to the diagnostic criteria of Simons et al.^7^ and Gerwin et al.^31^; next, four Styrofoam markers measuring 8 mm in diameter were used because of their isolating characteristic, positioned equidistantly at a distance of 25 mm from the center of the myofascial trigger point (Figure 1); after these procedures, the volunteers remained seated and at rest for 15 minutes in a room with controlled environment at a temperature of 22°±2°C and humidity of 50%, as established by Roy et al.^33^; and finally, three infrared images were sequentially captured at a distance of 100 cm from the subject and perpendicular to the myofascial trigger point^12^^,^ 21, thus allowing the muscle to be framed.

The room used for the thermographic examination was lit with fluorescent lamps, without the presence of electrical equipment generating heat and no incidence of sunlight or airflow on the volunteer^33^. The subjects were instructed to avoid taking a hot bath or shower, using topical agents such as creams or talc, practicing physical exercises, and ingesting stimulating substances such as caffeine, nicotine or chocolate during the two hours before data collection^19^^,^ 21.

During the collection procedures, the volunteers remained seated on a bench, with their trunk erect, hands on the thighs, and staring ahead. They were asked to let the region of the muscle being evaluated free of clothes or personal items, such as earrings or necklaces, in addition to keeping their hair tied up.

A thermal camera (T300, FLIR Systems, Wilsonville, OR, USA) was used to capture infrared images, operating with precision of up to 0.05 °C, emissivity of 0.98. The device was stabilized for 10 min prior to the reading.

### Analysis of infrared images

All analyses were conducted by using the QuickReport software, version 1.2 (FLIR Systems). Two examiners, who had previously received training with infrared thermography, performed the measurements of the images twice with a 1-week interval^34^, thus making it possible to assess the intra- and inter-rater reliability of the infrared image analyses.

**Figure 2.**
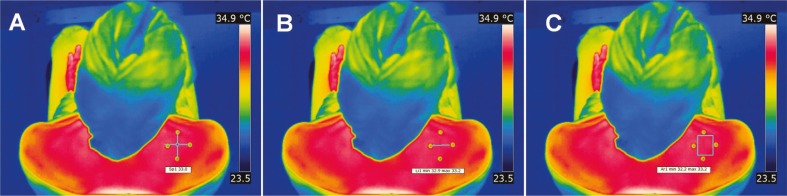
Analyses for point (A), line (B), and area (C) of the myofascial trigger point in the upper trapezius muscle.

Based on the analysis features of the software used in the present study, three forms of measurement of the skin temperature were performed over the myofascial trigger point: point analysis, in which the temperature of the central point of the area delimited by the markers was measured (Figure 2A); line analysis, in which a straight-line linking two markers was drawn across the region where the trigger point was located (Figure 2B); and area analysis, in which the area delimited by the four markers was established (Figure 2C). Initially, the mean values of skin temperature for the three analyses were calculated. Next, minimum and maximum values were considered for line and area analyses.

### Statistical analysis

Intraclass correlation coefficient (ICC_2,1_) was used to determine the intra- and inter-rater reliability, with its respective 95% confidence interval (CI 95%) , standard error of measurement (SEM), and minimum detectable change (MDC)^35^. Interpretation of ICC values was based on that suggested by Fleiss^22^. For values less than 0.40, the reliability was considered low; between 0.40 to 0.75, moderate; between 0.75 to 0.90, substantial; and finally, values greater than 0.90, excellent. All statistical analyses were performed using the SPSS software, version 17.0 (Chicago, IL, USA).

## Results

According to the eligibility criteria, twenty-eight volunteers were recruited from the university community. However, four volunteers were excluded from the study due to an NDI score of less than 5 points, thus resulting in a final sample of 24 subjects of both genders (23 females), 22 right-handed, mean age of 22.12 (SD=2.54) years, mean BMI of 21.04 (SD=1.95) kg/cm^2^, mean NDI score of 10.41 (SD=3.18) points, mean pain intensity of 5.00 (SD=1.66) points, and mean duration of cervical pain of 39.33 (SD=33.26) weeks.

Table 1 lists the values of intra-rater reliability, showing excellent reliability for point analysis (mean value), line analysis (mean and maximum values), and area analysis (mean, minimum, and maximum values), with ICC values ranging between 0.943 and 0.993. In addition, there was a moderate reliability for line analysis (minimum value), with ICC value equal to 0.591, and substantial reliability for area analysis (minimum value), with ICC value equal to 0.821. With respect to the SEM, there was variation in values between 0.13 and 1.57 °C. In turn, the MDC values ranged from 0.36 to 4.35 °C.

**Table 1.** Intra-rater reliability of the minimum, maximum, and mean values of skin temperature for point, line, and area analyses in the myofascial trigger point.

Analysis	Values	ICC	95% CI	SEM	MDC
Point	Mean	0.955^*^	0.928-0.972	0.34	0.94
Line	Minimum Maximum Mean	0.591^*^ 0.963^*^ 0.993^*^	0.346-0.744 0.942-0.977 0.989-0.996	1.57 0.28 0.13	4.35 0.78 0.36
Area	Minimum Maximum Mean	0.821^*^ 0.943^*^ 0.947^*^	0.714-0.888 0.909-0.964 0.915-0.967	0.66 0.32 0.16	1.83 0.89 0.44

ICC: Intra-class correlation coefficient; CI: Confidence interval; SEM: Standard error of measurement (in °C); MDC: Minimum detectable change (in °C).

* p<0.001.

Table 2 lists the values of inter-rater reliability, showing excellent reliability for point and line analysis (mean value), with ICC values equal to 0.908 and 0.918, respectively. For the other measures, there was moderate to substantial reliability, with ICC values ranging from 0.615 to 0.894. With respect to the SEM, there was variation in values between 0.43 and 1.22 °C. In turn, the MDC values ranged from 1.19 to 3.38 °C.

**Table 2.** Inter-rater reliability of the minimum, maximum, and mean values of skin temperature for point, line, and area analyses in the myofascial trigger point.

Analysis	Values	ICC	95% CI	SEM	MDC
Point	Mean	0.908^*^	0.853-0.942	0.48	1.33
Line	Minimum Maximum Mean	0.615^*^ 0.864^*^ 0.918^*^	0.348-0.759 0.783-0.915 0.869-0.949	1.22 0.52 0.43	3.38 1.44 1.19
Area	Minimum Maximum Mean	0.809^*^ 0.851^*^ 0.894^*^	0.695-0.880 0.762-0.907 0.831-0.934	0.62 0.50 0.44	1.72 1.39 1.22

ICC: Intra-class correlation coefficient; CI: Confidence interval; SEM: Standard error of measurement (in °C); MDC: Minimum detectable change (in °C).

* p<0.001.

## Discussion

In the present study, the intra- and inter-rater reliability of infrared image analyses by using point, line, and area approaches had substantial to excellent ICC values, except the minimum value for line analysis, as moderate ICC values were observed for intra- and inter-rate analyses.

The results of the present study are in partial accordance with those reported by Costa et al.^21^, who found excellent intra- and inter-rater reliability for point and line analyses regarding the masseter, temporalis anterior, suprahyoid, and upper trapezius muscles in individuals with or without temporomandibular disorder. However, it should be pointed out that these authors were not assessing myofascial trigger points in the skeletal muscles in question as their aim was to investigate skin temperature on the muscle belly.

Point analysis was also employed by Rodrigues-Bigaton et al.^36^ for assessment of skin temperature in the temporomandibular joint of individuals with and without arthralgia, with ICC values ranging from 0.841 to 0.874. Rodrigues-Bigaton et al.^37^ also used area analysis of the masseter and temporalis anterior muscle belly in both individuals with temporomandibular disorder and controls, reporting ICC values ranging from 0.945 to 0.998.

Some studies assessed the reliability of the infrared thermography in other clinical conditions, reporting results similar to those found in the present study. In the analysis of skin temperature regarding the paraspinal region, McCoy et al.^38^ found excellent intra- and inter-rater reliability. Choi et al.^39^ observed a high inter-rater reliability in the assessment of individuals with complex regional pain syndrome. In addition to these studies, Zaproudina et al.^40^ found high ICC values for inter-rater reliability in healthy subjects, however these authors identified reasonable ICC values when considering the temperature of the extremities on different days.

The studies conducted by Costa et al.^21^, Rodrigues-Bigaton et al.^36^, and Rodrigues-Bigaton et al.^37^ were based on the mean value of analyses performed for measurement of the skin temperature. Within this context, Klamann et al.^41^ assessed the intra-rater reliability of the temperature analysis of ocular surface, reporting ICC values of 0.947, 0.949, and 0.955 for minimum, maximum, and mean values, respectively. In the present study, not only the mean value was used but also minimum and maximum values of line and area analyses.

Regarding the values of SEM and MDC, published studies that evaluated the reliability of infrared thermography showed no such statistical measures^21^^,^ 37 ^-^ 40. In the present study, when considering the intra-rater reliability, higher SEM and MDC were observed for the minimum value of line (1.57 and 4.35 °C) and area (0.66 and 1.83) analyses. For inter-rater reliability, similar results were found, with higher SEM and MDC for the minimum value of the line (1.22 and 3.38 °C) and area (0.62 and 1.72 °C) analyses.

Thus, in general, mean (point, line, and area analyses) and maximum (line and area analyses) measures are the most reliable (intra-rater, ICC between 0.943 and 0.993; inter-rater, ICC between 0.851 and 0.918) and with less error (intra-rater, SEM between 0.13 and 0.34 °C, and MDC between 0.36 and 0.94 °C; inter-rater, SEM between 0.43 and 0.52 °C, and MDC between 1.19 and 1.44 °C). Moreover, in a more rigorous analysis of ICC values, considering the lower limit of the CI 95% and excepting the minimum values of the area and line analyzes, excellent intra-rater reliability (ICC values between 0.909 and 0.989) and substantial inter-rater reliability (ICC values between 0.762 and 0.869) were observed. These results give more robustness to the applicability of the methods of analyses (mean and maximum values) of the infrared images.

Considering the relevance of SEM and MDC in reliability studies, Tucci et al.^35^ evaluated a specific test for identification of shoulder impingement syndrome and also found that previously published studies in the same subject did not consider these statistical measures in reliability analysis. In addition, these authors emphasize the importance of knowing the values for SEM and MDC as these numbers give a good indication of the minimal score difference between evaluations that could be considered as real improvement.

Finally, infrared thermography has been employed for the evaluation of different musculoskeletal conditions^42^^-^ 44. Therefore, the aim of the present study was to standardize the infrared image analyses of myofascial trigger points, thus making it possible to support the use of infrared thermography in clinical practice and research for either mapping the skin temperature of a given site or even for assessing the effects of therapeutic resources in musculoskeletal dysfunctions^4^^,^ 6 ^,^ 12.

The present study had the limitation of not including volunteers with latent myofascial trigger points, since these differ from the active ones due to the presence of algesic substances, among other features^3^. Moreover, we suggest that future studies assess the reliability of the entire procedure of collecting thermographic data: patient preparation, instrumentation, recording, and analysis of the infrared images.

## Conclusion

The methods of infrared image analysis of myofascial trigger points in the upper trapezius muscle proposed by the present study showed acceptable intra- and inter-rater reliability values, which supports the use of these methodologies in clinical and research practices.

## References

[1] Ge HY, Arendt-Nielsen L (2011). Latent myofascial trigger points. Curr Pain Headache Rep.

[2] Bron C, Dommerholt JD (2012). Etiology of myofascial trigger points. Curr Pain Headache Rep.

[3] Shah JP, Gilliams EA (2008). Uncovering the biochemical milieu of myofascial trigger
points using in vivo microdialysis: an application of muscle pain concepts to
myofascial pain syndrome. J Bodyw Mov Ther.

[4] Montañez-Aguilera FJ, Valtueña-Gimeno N, Pecos-Martín D, Arnau-Masanet R, Barrios-Pitarque C, Bosch-Morell F (2010). Changes in a patient with neck pain after application of
ischemic compression as a trigger point therapy. J Back Musculoskelet Rehabil.

[5] Alonso-Blanco C, Fernández-de-las-Peñas C, Fernández-Mayoralas DM, de-la-Llave-Rincón AI, Pareja JA, Svensson P (2011). Prevalence and anatomical localization of muscle
referred pain from active trigger points in head and neck musculature in adults
and children with chronic tension-type headache. Pain Med.

[6] Tekin L, Akarsu S, Durmuş O, Cakar E, Dinçer U, Kıralp MZ (2013). The effect of dry needling in the treatment of
myofascial pain syndrome: a randomized double-blinded placebo-controlled
trial. Clin Rheumatol.

[7] Simons DG, Travell J, Simons LS (1999). Myofascial pain and dysfunction: the trigger point
manual.

[8] Thomas K, Shankar H (2013). Targeting myofascial taut bands by
ultrasound. Curr Pain Headache Rep.

[9] Sikdar S, Ortiz R, Gebreab T, Gerber LH, Shah JP (2010). Understanding the vascular environment of myofascial
trigger points using ultrasonic imaging and computational modeling. Conf Proc IEEE Eng Med Biol Soc.

[10] Ballyns JJ, Shah JP, Hammond J, Gebreab T, Gerber LH, Sikdar S (2011). Objective sonographic measures for characterizing
myofascial trigger points associated with cervical pain. J Ultrasound Med.

[11] Ibarra JM, Ge HY, Wang C, Martínez Vizcaíno V, Graven-Nielsen T, Arendt-Nielsen L (2011). Latent myofascial trigger points are associated with an
increased antagonistic muscle activity during agonist muscle
contraction. J Pain.

[12] Hakgüder A, Birtane M, Gürcan S, Kokino S, Turan FN (2003). Efficacy of low level laser therapy in myofascial pain
syndrome: an algometric and thermographic evaluation. Lasers Surg Med.

[13] Haddad DS, Brioschi ML, Arita ES (2012). Thermographic and clinical correlation of myofascial
trigger points in the masticatory muscles. Dentomaxillofac Radiol.

[14] Szentkuti A, Kavanagh HS, Grazio S (2011). Infrared thermography and image analysis for biomedical
use. Period Biol.

[15] Brioschi ML, Macedo JF, Macedo RAC (2003). Skin thermometry: new concepts. J Vasc Bras.

[16] Holey LA, Dixon J, Selfe J (2011). An exploratory thermographic investigation of the
effects of connective tissue massage on autonomic function. J Manipulative Physiol Ther.

[17] Gratt BM, Sickles EA, Ross JB, Wexler CE, Gornbein JA (1994). Thermographic assessment of craniomandibular disorders:
diagnostic interpretation versus temperature measurement analysis. J Orofac Pain.

[18] Kontos M, Wilson R, Fentiman I (2011). Digital infrared thermal imaging (DITI) of breast
lesions: sensitivity and specificity of detection of primary breast
cancers. Clin Radiol.

[19] Dibai AV, Packer AC, Costa AC, Berni-Schwarzenbeck KC, Rodrigues-Bigaton D (2012). Assessment of the upper trapezius muscle temperature in
women with and without neck pain. J Manipulative Physiol Ther.

[20] Dibai-Filho AV, Costa AC, Packer AC, Rodrigues-Bigaton D (2013). Correlation between skin surface temperature over
masticatory muscles and pain intensity in women with myogenous temporomandibular
disorder. J Back Musculoskelet Rehabil.

[21] Costa AC, Dibai AV, Packer AC, Rodrigues-Bigaton D (2013). Intra and inter-rater reliability of infrared image
analysis of masticatory and upper trapezius muscles in women with and without
temporomandibular disorder. Braz J Phys Ther.

[22] Fleiss J (1986). The design and analysis of clinical experiments.

[23] Bonett DG (2002). Sample size requirements for estimating intraclass
correlations with desired precision. Stat Med.

[24] Muñoz-Muñoz S, Muñoz-García MT, Alburquerque-Sendín F, Arroyo-Morales M, Fernández-de-las-Peñas C (2012). Myofascial trigger points, pain, disability, and sleep
quality in individuals with mechanical neck pain. J Manipulative Physiol Ther.

[25] Bogduk N (2003). The anatomy and pathophysiology of neck
pain. Phys Med Rehabil Clin N Am.

[26] Cook C, Richardson JK, Braga L, Menezes A, Soler X, Kume P (2006). Cross-cultural adaptation and validation of the
Brazilian Portuguese version of the Neck Disability Index and Neck Pain and
Disability Scale. Spine (Phila Pa 1976).

[27] Vernon H, Mior S (1991). The Neck Disability Index: a study of reliability and
validity. J Manipulative Physiol Ther.

[28] Ferreira-Valente MA, Pais-Ribeiro JL, Jensen MP (2011). Validity of four pain intensity rating
scales. Pain.

[29] Oliveira-Campelo NM, de Melo CA, Alburquerque-Sendín F, Machado JP (2013). Short- and medium-term effects of manual therapy on
cervical active range of motion and pressure pain sensitivity in latent myofascial
pain of the upper trapezius muscle: a randomized controlled trial. J Manipulative Physiol Ther.

[30] Ziaeifar M, Arab AM, Karimi N, Nourbakhsh MR (2014). The effect of dry needling on pain, pressure pain
threshold and disability in patients with a myofascial trigger point in the upper
trapezius muscle. J Bodyw Mov Ther.

[31] Gerwin RD, Shannon S, Hong CZ, Hubbard D, Gevirtz R (1997). Interrater reliability in myofascial trigger point
examination. Pain.

[32] Alburquerque-Sendín F, Camargo PR, Vieira A, Salvini TF (2013). Bilateral myofascial trigger points and pressure pain
thresholds in the shoulder muscles in patients with unilateral shoulder
impingement syndrome: a blinded, controlled study. Clin J Pain.

[33] Roy RA, Boucher JP, Comtois AS (2006). Digitized infrared segmental thermometry: time
requirements for stable recordings. J Manipulative Physiol Ther.

[34] Van Maanen CJ, Zonnenberg AJ, Elvers JW, Oostendorp RA (1996). Intra/interrater reliability of measurements on body
posture photographs. Cranio.

[35] Tucci H, Martins J, Sposito G, Camarini P, Oliveira A (2014). Closed Kinetic Chain Upper Extremity Stability test
(CKCUES test): a reliability study in persons with and without shoulder
impingement syndrome. BMC Musculoskelet Disord.

[36] Rodrigues-Bigaton D, Dibai AV, Costa AC, Packer AC, de Castro EM (2013). Accuracy and reliability of infrared thermography in the
diagnosis of arthralgia in women with temporomandibular disorder. J Manipulative Physiol Ther.

[37] Rodrigues-Bigaton D, Dibai-Filho AV, Packer AC, Costa AC, de Castro EM (2014). Accuracy of two forms of infrared image analysis of the
masticatory muscles in the diagnosis of myogenous temporomandibular
disorder. J Bodyw Mov Ther.

[38] McCoy M, Campbell I, Stone P, Fedorchuk C, Wijayawardana S, Easley K (2011). Intra-examiner and inter-examiner reproducibility of
paraspinal thermography. PLoS One.

[39] Choi E, Lee PB, Nahm FS (2013). Interexaminer reliability of infrared thermography for
the diagnosis of complex regional pain syndrome. Skin Res Technol.

[40] Zaproudina N, Varmavuo V, Airaksinen O, Närhi M (2008). Reproducibility of infrared thermography measurements in
healthy individuals. Physiol Meas.

[41] Klamann MK, Maier AK, Gonnermann J, Klein JP, Pleyer U (2012). Measurement of dynamic ocular surface temperature in
healthy subjects using a new thermography device. Curr Eye Res.

[42] Dibai AV, Packer AC, Costa AC, Rodrigues-Bigaton D (2013). Accuracy of infrared thermography of the masticatory
muscles for the diagnosis of myogenous temporomandibular disorder. J Manipulative Physiol Ther.

[43] Zaproudina N, Airaksinen O, Närhi M (2013). Are the infrared thermography findings skin
temperature-dependent? a study on neck pain patients. Skin Res Technol.

[44] Roy RA, Boucher JP, Comtois AS (2013). Comparison of paraspinal cutaneous temperature
measurements between subjects with and without chronic low back
pain. J Manipulative Physiol Ther.

